# Long-term outcomes of laparoscopic liver resection versus open liver resection for hepatocellular carcinoma: A single-center 10-year experience

**DOI:** 10.3389/fonc.2023.1112380

**Published:** 2023-01-25

**Authors:** Feng Tian, Songyao Leng, Jian Chen, Yong Cao, Li Cao, Xiaojun Wang, Xuesong Li, Juan Wang, Shuguo Zheng, Jianwei Li

**Affiliations:** ^1^ Department of Hepatobiliary Surgery, Southwest Hospital, Army Medical University, Chongqing, China; ^2^ Department of General Surgery, The First People’s Hospital of Neijiang, Neijiang, Sichuan, China; ^3^ Clinical Skills Training Center, Southwest Hospital, Army Medical University, Chongqing, China

**Keywords:** hepatocellar carcinoma, laparoscopic liver resection, open liver resection, laparoscopic minor liver resection in difficult segments, laparoscopic major hepatectomy, prognosis

## Abstract

**Background:**

Laparoscopic liver resection (LLR) for hepatocellular carcinoma (HCC) has increased. However, the long-term outcomes of LLR for HCCs should be validated further. Besides, the validity of laparoscopic minor liver resection in difficult segments (1, 4a, 7, 8) (LMLR-DS) and laparoscopic major hepatectomy (LMH) for HCCs need to be studied.

**Methods:**

A total of 1773 HCC patients were collected: 683 received LLR and 1090 received OLR. Propensity score matching (PSM) with 1:1 ratio was used to eliminate the selection bias. Short-term and long-term outcomes were compared. In subgroup analyses, the validity of LMLR-DS or LMH for HCCs was studied.

**Results:**

After PSM, 567 patients were in LLR or OLR group. LLR had lower intraoperative blood-loss and shorter postoperative hospital-stays than OLR. The postoperative complications were lower in LLR group (23.8% *vs.* 32.8%, P=0.001). The Overall survival (OS) and disease-free survival (DFS) had no significant difference between LLR and OLR groups (P=0.973, P=0.812). The cumulative 1-, 3-, and 5-year OR rates were 87.9%, 68.9%, and 57.7% for LLR group, and 85.9%, 68.8%, 58.8% for OLR group. The cumulative 1-, 3-, and 5-year DFS rates were 73.0%, 51.5%, 40.6% for LLR group, and 70.3%, 49.0%, 42.4% for OLR group. In subgroup analyses, 178 patients were in LMLR-DS or open surgery (OMLR-DS) group after PSM. LMLR-DS had lower intraoperative blood-loss and shorter postoperative hospital-stays than OMLR-DS. The postoperative complications were lower in LMLR-DS group. The OS and DFS had no difference between LMLR-DS and OMLR-DS groups. The cumulative 5-year OR and DFS rates were 61.6%, 43.9% for LMLR-DS group, and 66.5%, 47.7% for OMLR-DS group. In another subgroup analyses, 115 patients were in LMH or open major hepatectomy (OMH) group. LMH had lower blood-loss and shorter postoperative hospital-stays than OMH. The complications, OS and DFS had no significantly differences between two groups. The cumulative 5-year OR and DFS rates were 44.3%, 29.9% for LMH group, and 44.7%, 33.2% for OMH group.

**Conclusions:**

LLR for HCCs showed better short-term outcomes and comparable long-term outcomes with OLR, even for patients who received LMLR-DS or LMH. LLR could be reliable and recommended for HCC treatment.

## Introduction

The Global Cancer Statistics 2020 reports that primary liver cancer is the sixth common malignancy and the third leading cause of tumor-related death (1). Hepatocellular carcinoma (HCC), which accounts for 75-85% of primary liver cancer, is a global health challenge (1, 2). Liver resection (LR) remains the mainstay of curative treatment for HCC (3, 4). LR mainly includes two types: open liver resection (OLR) and laparoscopic liver resection (LLR). OLR is the traditional and standard procedure for HCC treatment. With the development of laparoscopic technique and equipment, LLR has been progressively increasing in recent years (5–7).

Clinical researches of LLR for HCCs have always been the hot area worldwide. Advantages of LLR are reported with regard to improved short-term outcomes compared with OLR (8). However, the long-term outcome of LLR for HCCs has become an important topic of debate (9–12). Although several recent studies show that LLR has similar long-term outcomes with OLR for HCCs (13–16), it needs to be validated further in more studies with a larger number of cases. Moreover, according to Asia Pacific Consensus and Southampton Consensus Guidelines (6, 7), laparoscopic minor liver resections (LMLR) in anterolateral segments (2, 3, 4b, 5, 6), is reliable and recommended for HCC treatment, but on the other hand, laparoscopic minor liver resection in difficult segments (1, 4a, 7, 8) (LMLR-DS) and laparoscopic major hepatectomy (LMH) for HCC treatment needs to be investigated further.

The aim of this study was to compare the short-term and long-term outcomes of LLR with those of OLR for HCC in well-matched patient groups using propensity score matching (PSM) with a large number of cases at a single center. Moreover, the outcomes of LMLR-DS and LMH for HCCs were studied in subgroup analyses.

## Methods

### Study design and patients

We retrospectively reviewed the data for patients who received LR for HCC in Southwest hospital, Army medical university, Chongqing, China from January 2009 to December 2017.The inclusion criteria was as follows: (1) patients aged 18-75 years; (2) liver function classified as Child-Pugh A or B; (3) the remaining liver volume was adequate with the preoperative evaluation; (4) histopathological confirmation of HCC; and (5) no extrahepatic metastasis. The exclusion criteria were as follows: (1) patients with recurrent HCC; (2) liver resection combined with abdominal organ resection other than gallbladder resection; and (3) patients with HCC who underwent prior RFA or TACE.

A total of 1773 patients with HCC were collected in this study: 683 patients in LLR group and 1090 patients in OLR group (**Supplementary Table S1**). Preoperative evaluations were similar in two groups and included routine blood tests, liver function, coagulation examinations, serum tumor markers, indocyanine green retention test at 15 minutes (ICG-R15), and triphasic enhanced computed tomography (CT) and/or magnetic resonance imaging (MRI). Routine blood and hepatic function tests were performed after surgery. Abdominal ultrasonography was routinely conducted for patients before discharge. LMLR (≤ 2 segments) or LMH (≥ 3 contiguous segments) were defined according to the previous study (8).The severity of postoperative complications was graded by the Clavien–Dindo classification, and the severe complications were defned as Clavien–Dindo grade III and above (17). This study was approved by the Ethics Committee of Southwest Hospital of Army Medical University.

### Surgical procedure

For LLR, either 3D or 2D laparoscopic system was used. The patient was placed in the supine position. Carbon dioxide pneumoperitoneum was established with a pressure of 11–13 mmHg. Two 12 mm ports and two 5 mm ports were applied for the operation, and one 12mm port was used for the laparoscope. Laparoscopic ultrasonography was routinely performed to confirm the positions of tumors and guide the dissection line. The Pringle maneuver was applied to control blood loss. The liver parenchyma was dissected using the ultrasonic dissector. Intraparenchymal vascular and biliary structures (≥3 mm) were ligated by titanium clips or Hem-o-lock clips. The main Glissonian pedicles or hepatic veins were transected by the laparoscopic linear stapler or Hem-o-lock clips after ligation. The specimen was placed into a sterile bag and extracted through a suprapubic incision or an upper abdominal midline incision.

For OLR, a reverse L-incision or right subcostal incision was conducted. The operating procedure was similar to LLR. CUSA or clamp crushing was used as the main method for liver parenchyma dissection.

### Follow-up

The follow-up was conducted every one month within three months after operation, and then every three months within two years and three or six months afterwards. Routine investigations at each follow-up included routine blood tests, liver function, tumor markers, abdominal ultrasonography, and even CT or MRI if necessary. Overall survival (OS) was defined as the time from the surgery date to death from any cause or the last follow-up. Disease free survival (DFS) was defined as the time from the surgery date to tumor recurrence. All patients were followed up with the same protocol.

### Statistical analysis

PSM with 1:1 ratio was used to eliminate the selection bias between LLR and OLR groups based on the nearest neighbor matching method without replacement. The propensity score covariates in this study included age, gender, HBV, HCV, liver cirrhosis, Child–Pugh score, American Society of Anesthesiologists score (ASA score), preoperative blood tests (ALT, TBIL, ALB, PT, Platelet count and AFP), ICG-R15, tumor location, tumor number, largest tumor diameter, type of LR, range of LR, resection tumor margin, margin status, histological grade, satellite nodule, portal vein invasion, bile duct invasion and TNM stage (**Table 1**). After matching, P values for the group samples were all greater than 0.05, indicating a good balance. Continuous variables were compared using a t test or Mann–Whitney test. Categorical variables were compared using the χ^2^ or Fisher exact test. A two-tailed P value <0.05 was considered significant. Survival curves were estimated by the Kaplan-Meier method for OS and DFS, the log-rank test was used for between-group comparisons. P values < 0.05 were considered significant. All statistical analyses were performed using SPSS software version 25.0 (IBM SPSS Inc. Chicago, IL).

**Table 1:** Baseline patient characteristics between laparoscopic liver resection (LLR) and open liver resection (OLR) groups.

Characteristics	Before PSM			After PSM		
	LLR (N=683)	OLR (N=1090)	P	LLR (N=567)	OLR (N=567)	P
**Age**	51.00 (45.00-60.00)	48.00 (42.00-57.00)	<0.001*	50.00 (44.00-59.00)	50.00 (44.00-60.00)	0.643
**Gender**			0.245			0.135
Male	586 (85.8%)	956 (87.7%)		484 (85.4%)	501 (88.4%)	
Female	97 (14.2%)	134 (12.3%)		83 (14.6%)	66 (11.6%)	
**Positive HBV-DNA**	587 (85.9%)	980 (89.9%)	0.011*	495 (87.3%)	502 (88.5%)	0.524
**Positive HCV-RNA**	8 (1.2%)	10 (0.9%)	0.604	6 (1.1%)	4 (0.7%)	0.525
**Liver cirrhosis**	449 (65.7%)	717 (65.8%)	0.986	379 (66.8%)	382 (67.4%)	0.850
**Child-Pugh score**			0.060			1.000
A	682 (99.9%)	1080 (99.1%)		566 (99.8%)	566 (99.8%)	
B	1 (0.1%)	10 (0.9%)		1 (0.2%)	1 (0.2%)	
**ASA score**			0.023*			0.953
I	372 (54.5%)	533 (48.9%)		302 (53.3%)	301 (53.1%)	
II	311 (45.5%)	557 (51.1%))		265 (46.7%)	266 (46.9%)	
**TBIL(µmol/L)**	15.20 (11.70-19.10)	16.10 (12.70-20.10)	<0.001*	15.30 (11.70-19.00)	15.30 (12.20-19.50)	0.268
**ALT (IU/L)**	34.00 (24.00-48.00)	38.00 (27.00-55.00)	<0.001*	35.00 (24.70-50.20)	36.00 (25.90-51.00)	0.409
**ALB**	42.70 (39.60-45.10)	42.90 (39.70-45.70)	0.105	42.80 (39.70-45.10)	42.90 (39.60-45.60)	0.620
**PT (INR)**	1.01 (0.97-1.06)	1.02 (0.98-1.08)	<0.001*	1.02 (0.97-1.06)	1.01 (0.97-1.06)	0.214
**Platelet count** (*103/μL)	134.00 (96.00-172.00)	144.00 (106.75-195.00)	<0.001*	134.00 (96.00-173.00)	134.00 (102.00-176.00)	0.294
**AFP (≥400 ng/mL)**	186 (27.2%)	433 (39.7%)	<0.001*	174 (30.7%)	171 (30.2%)	0.846
**ICG-R15 (%)**	4.40 (2.70-6.90)	4.50 (2.60-7.20)	0.465	4.50 (2.70-7.00)	4.40 (2.50-6.80)	0.623
**Tumor Location**			0.011*			0.754
Difficult segments (1, 4a, 7, 8)	205 (30.0%)	391 (35.9%)		195 (34.4%)	190 (33.5%)	
Simple segments (2, 3, 4b, 5, 6)	478 (70.0%)	699 (64.1%)		372 (65.6%)	377 (66.5%)	
**Tumor number**			0.712			0.767
1	637 (93.3%)	1006 (92.3%)		524 (92.4%)	520 (91.7%)	
2-3	42 (6.1%)	78 (7.1%)		39 (6.9%)	44 (7.8%)	
≥4	4 (0.6%)	6 (0.6%)		4 (0.7%)	3 (0.5%)	
**Largest tumor diameters**			<0.001*			0.892
≤5cm	535 (78.3%)	553 (50.7%)		419 (73.9%)	421 (74.3%)	
>5cm	148 (21.7%)	537 (49.3%)		148 (26.1%)	146 (25.7%)	
**Type of LR**			0.272			0.905
Anatomical LR	319 (46.7%)	480 (44.0%)		255 (45.0%)	257 (45.3%)	
Non-anatomical LR	364 (53.3%)	610 (56.0%)		312 (55.0%)	310 (54.7%)	
**Range of LR**			<0.001*			0.717
Major	124 (18.2%)	380 (34.9%)		119 (21.0%)	124 (21.9%)	
Minor	559 (81.8%)	710 (65.1%)		448 (79.0%)	443 (78.1%)	
**Resection tumor margin**			0.054			0.307
≥1cm	658 (96.3%)	1028 (94.3%)		546 (96.3%)	539 (95.1%)	
<1cm	25 (3.7%)	62 (5.7%)		21 (3.7%)	28 (4.9%)	
**Margin status**			0.260			1.000
Negative	682 (99.9%)	1084 (99.4%)		566 (99.8%)	566 (99.8%)	
Positive	1 (0.1%)	6 (0.6%)		1 (0.2%)	1 (0.2%)	
**Histological grade**			<0.001*			0.077
Low	86 (12.6%)	219 (20.1%)		72 (12.7%)	96 (16.9%)	
Moderate	546 (79.9%)	811 (74.4%)		459 (81.0%)	428 (75.5%)	
High	51 (7.5%)	60 (5.5%)		36 (6.3%)	43 (7.6%)	
**Satellite nodule**			<0.001*			0.463
Positive	7 (1.0%)	42 (3.9%)		7 (1.2%)	10 (1.8%)	
Negative	676 (99.0%)	1048 (96.1%)		560 (98.8%)	557 (98.2%)	
**Portal vein invasion**			<0.001*			0.316
Positive	22 (3.2%)	136 (12.5%)		22 (3.9%)	29 (5.1%)	
Negative	661 (96.8%)	954 (87.5%)		545 (96.1%)	538 (94.9%)	
**Bile duct invasion**			0.655			1.000
Positive	1 (0.1%)	4 (0.4%)		1 (0.2%)	1 (0.2%)	
Negative	682 (99.9%)	1086 (99.6%)		566 (99.8%)	566 (99.8%)	
**TNM stage**			<0.001*			0.359
I-II	646 (94.6%)	918 (84.2%)		530 (93.5%)	522 (92.1%)	
III-IV	37 (5.4%)	172 (15.8%)		37 (6.5%)	45 (7.9%)	

HBV, hepatitis B virus; HCV, hepatitis C virus; ASA American Society of Anesthesiologists;TBIL, total bilirubin; ALT, alanine transaminase; PT, prothrombin time; AFP,alpha-fetoprotein; ICG-R15, indocyanine green retention test at 15 minutes. *P < 0.05.

(*P < 0.05, statistical significance).

## Results

### Baseline characteristics

A total of 1773 patients with HCC were collected in this study, 683 patients received LLR and 1090 patients received OLR. The baseline characteristics were shown in **Table 1**. Between LLR and OLR groups, age, HBV-DNA and ASA scores were significantly different. Besides, the preoperative serum levels of TBIL, ALT, PT, Platelet count and AFP were different. Among the operative characteristics, tumor location, largest tumor diameters and the range of LR were significantly different between two groups. The characteristics of histological grade and portal vein invasion were also significantly different. Moreover, the TNM stage was different between two groups. After PSM with 1:1 ratio, there were 567 patients in each group with well-balanced baseline characteristics (**Table 1**).

### Short-term outcomes

Short-term outcomes were compared between LLR and OLR groups after PSM (**Table 2**). The operative time was similar between two groups. On the other hand, the operative blood loss and the rate of blood transfusion in LLR groups were significantly lower than them in OLR group (200.00 ml *vs.* 300.00 ml, P<0.001; 8.6% *vs.* 12.3%, P=0.042). The perioperative mortality was similar between two groups. However, the overall postoperative complications in LLR group were significantly lower than them in OLR group (23.8% *vs.* 32.8%, P=0.001). The complications mainly included seroperitoneum, hydrothorax, infection, hemorrhage, bile leak, liver failure, respiratory failure and renal failure. Moreover, the severe complications (Clavien–Dindo grade III and above) were also significantly lower in LLR groups (5.1% *vs.* 8.6%, P=0.019). Besides, the postoperative hospital stays were significantly shorter in LLR group (10.00 days *vs.* 13.00 days, P<0.001). Together, the results showed that patients in LLR group had better short-term outcomes compared with them in OLR group.

**Table 2:** Operative details and postoperative outcomes between laparoscopic liver resection (LLR) and open liver resection (OLR) groups after propensity score matching.

	LLR (N=567)	OLR (N=567)	P
**Operative time (min)**	205.00 (150.00-267.00)	200.00 (160.00-250.00)	0.652
**Blood loss (ml)**	200.00 (150.00-400.00)	300.00 (200.00-400.00)	<0.001*
**Blood transfusion**	49 (8.6%)	70 (12.3%)	0.042*
**Perioperative mortality**	0 (0%)	0 (0%)	1.000
**Overall complications**	135 (23.8%)	186 (32.8%)	0.001*
Seroperitoneum	55 (9.7%)	73 (12.9%)	
Hydrothorax	37 (6.5%)	47 (8.3%)	
Infection	25 (4.4%)	35 (6.2%)	
Hemorrhage	24 (4.2%)	51 (9.0%)	
Bile leak	3 (0.5%)	7 (1.2%)	
Liver failure	2 (0.4%)	4 (0.7%)	
Respiratory failure	1 (0.2%)	4 (0.7%)	
Renal Failure	0 (0.0%)	1 (0.2%)	
**Severe complications** **(Clavien–Dindo III -IV)**	29 (5.1%)	49 (8.6%)	0.019*
**Postoperative hospital stay (D)**	10.00 (8.00-12.00)	13.00 (11.00-15.00)	<0.001*

(*P < 0.05, statistical significance).

### Long-term outcomes

**Figure 1:**
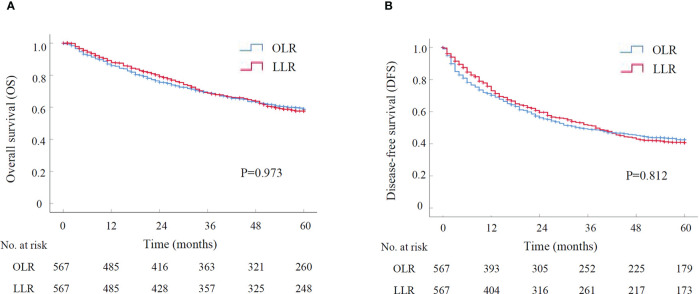
The survival curve of LLR versus OLR for HCCs after PSM by Kaplan-Meier analysis (N=567 for each group). **(A)** Overall survival; **(B)** Disease-free survival. LLR, laparoscopic liver resection; OLR, open liver resection; PSM, propensity score matching.

The long-term outcomes were compared between LLR and OLR groups after PSM. The OS and DFS curves were presented in **Figure 1**. Survival analysis showed that the OS had no significant difference between LLR and OLR groups (**Figure 1**, P=0.973). The cumulative 1-, 3-, and 5-year OS rates were 87.9%, 68.9%, and 57.7% for patients in LLR group, and were 85.9%, 68.8%, and 58.8% for patients in OLR group, respectively. Consistently, the DFS had no difference between LLR and OLR groups (**Figure 1**, P=0.812). The cumulative 1-, 3-, and 5-year DFS rates were 73.0%, 51.5%, and 40.6% for patients in LLR group, and were 70.3%, 49.0%, and 42.4% for patients in OLR group, respectively. Together, the results showed that patients in LLR group had comparable long-term outcomes with them in OLR group.

### Subgroup analysis: LMLR-DS versus open minor liver resection in difficult segments (1, 4a, 7 and 8) (OMLR-DS) for HCCs

**Figure 2:**
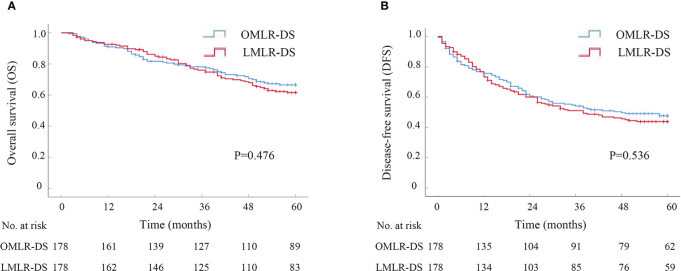
The survival curve of LMLR-DS versus OMLR-DS for HCCs after PSM by Kaplan-Meier analysis (N=178 for each group). **(A)** Overall survival; **(B)** Disease-free survival. LMLR-DS, laparoscopic minor liver resection in difficult segments (1, 4a, 7 and 8); OMLR-DS, open minor liver resection in difficult segments; PSM, propensity score matching.

A subgroup analysis was performed to assess the outcomes of LMLR-DS for HCCs compared with OMLR-DS. After PSM with 1:1 ratio, there were 178 patients in LMLR-DS or OMLR-DS group, and all baseline characteristics were well-balanced (**Supplementary Table S2**). The operative time and the rate of blood transfusion were similar in two groups. While, the operative blood loss in LMLR-DS group was significantly lower than it in OMLR-DS group (200.00 ml *vs.* 300.00 ml, P<0.001). There was no perioperative death in both two groups. However, the overall and severe postoperative complications were significantly lower in LMLR-DS group compared with them in OMLR-DS group (27.5% *vs.* 39.9%, P=0.014; 3.9% *vs.* 11.2%, P=0.009; **Table 3**). The postoperative stays were significantly shorter in LMLR-DS group (10.00 days *vs.* 13.50 days, P<0.001). For long-term survival analysis, the OS had no significant difference between LMLR-DS and OMLR-DS groups (P=0.476, **Figure 2**). The cumulative 1-, 3-, and 5-year OS rates were 92.6%, 76.0%, and 61.6% for patients in LMLR-DS group, and were 90.9%, 78.2%, and 66.5% for patients in OMLR-DS group, respectively. Consistently, the DFS had no difference between LMLR-DS and OMLR-DS groups (**Figure 2**, P=0.536). The cumulative 1-, 3-, and 5-year DFS rates were 76.7%, 51.2%, and 43.9% for patients in LMLR-DS group, and were 75.7%, 54.1%, and 47.7% for patients in OMLR-DS group, respectively. Together, the results showed that LMLR-DS showed better short-term outcomes and similar long-term outocomes with OMLR-DS for HCCs.

**Table 3:** Operative details and postoperative outcomes between laparoscopic minor liver resection in difficult segments (LMLR-DS) and open minor liver resection in difficult segments (OMLR-DS) groups after propensity score matching.

	LMLR-DS (N=178)	OMLR-DS (N=178)	P
**Operative time (min)**	202.50 (150.00-300.25)	204.50 (160.00-254.00)	0.961
**Blood loss (ml)**	200.00 (150.00-400.00)	300.00 (200.00-500.00)	<0.001*
**Blood transfusion**	17 (9.6%)	23 (12.9%)	0.314
**Perioperative mortality**	0 (0%)	0 (0%)	1.000
**Overall complications**	49 (27.5%)	71 (39.9%)	0.014*
Seroperitoneum	21 (11.8%)	30 (16.9%)	
Hydrothorax	16 (9.0%)	26 (14.6%)	
Infection	7 (3.9%)	9 (5.1%)	
Hemorrhage	7 (3.9%)	15 (8.4%)	
Bile leak	0 (0.0%)	2 (1.1%)	
Renal Failure	0 (0.0%)	1 (0.6%)	
**Severe complications** **(Clavien–Dindo III -IV)**	7 (3.9%)	20 (11.2%)	0.009*
**Postoperative hospital stay (D)**	10.00 (8.00-12.00)	13.50 (12.00-15.00)	<0.001*

(*P < 0.05, statistical significance).

### Subgroup analysis: LMH versus open major hepatectomy (OMH) for HCCs

**Figure 3:**
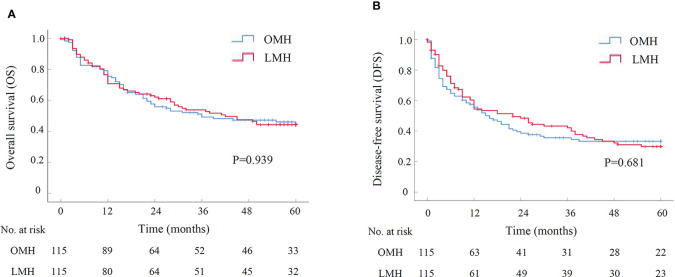
The survival curve of LMH versus OMH for HCCs after PSM by Kaplan-Meier analysis (N=115 for each group). **(A)** Overall survival; **(B)** Disease-free survival. LMH, laparoscopic major hepatectomy; OMH, open major hepatectomy; PSM, propensity score matching.

Another subgroup analysis was performed to assess short-term and long-term outcomes of LMH for HCCs compared with OMH. There were 115 patients in either LMH or OMH group after PSM, and all baseline characteristics were well-balanced (**Supplementary Table S3**). The operative time was similar in two groups. However, the operative blood loss and the rate of blood transfusion in LMH group were significantly lower than them in OMH group (300.00ml *vs.* 400.00ml, P=0.003; 13.0% *vs.* 24.3%, P=0.028; **Table 4**). There was no perioperative death in both two groups. Besides, the overall and severe postoperative complications had no significant difference between two groups (**Table 4**). The postoperative hospital stays were significantly shorter in LMH group (11.00 days *vs.* 14.00 days, P<0.001). For long-term survival analyses, the OS had no significant difference between LMH and OMH groups (P=0.939, **Figure 3**). The cumulative 1-, 3-, and 5-year OS rates were 70.8%, 53.9%, and 44.3% for patients in LMH group, and were 75.5%, 49.2%, and 44.7% for patients in OMH group, respectively. Consistently, the DFS had no difference between two groups (P=0.681, **Figure 3**). The cumulative 1-, 3-, and 5-year DFS rates were 54.4%, 42.2%, and 29.9% for patients in LMH group, and were 55.7%, 33.5%, and 33.2% for patients in OMH group, respectively. Together, the results showed that LHM for HCCs had comparable short-term and long-term outcomes with OHM.

**Table 4:** Operative details and postoperative outcomes between laparoscopic major hepatectomy (LMH) and open major hepatectomy (OMH) groups after propensity score matching.

	LMH (N=115)	OMH (N=115)	P
**Operative time (min)**	255.00 (204.00-321.00)	253.00 (203.00-315.00)	0.797
**Blood loss (ml)**	300.00 (200.00-500.00)	400.00 (300.00-600.00)	0.003*
**Blood transfusion**	15 (13.0%)	28 (24.3%)	0.028*
**Perioperative mortality**	0 (0%)	0 (0%)	1.000
**Overall complications**	34 (29.6%)	44 (38.3%)	0.164
Seroperitoneum	12 (10.4%)	14 (12.2%)	
Hydrothorax	10 (8.7%)	9 (7.8%)	
Hemorrhage	8 (7.0%)	17 (14.8%)	
Infection	4 (3.5%)	11 (9.6%)	
Bile leak	3 (2.6%)	2 (1.7%)	
Liver failure	2 (1.7%)	2 (1.7%)	
Respiratory failure	0 (0.0%)	1 (0.9%)	
**Severe complications** **(Clavien–Dindo III -IV)**	7 (6.1%)	12 (10.4%)	0.231
**Postoperative hospital stay (D)**	11.00 (9.00-13.00)	14.00 (12.00-17.00)	<0.001*

(*P < 0.05, statistical significance).

## Discussion

As a minimally invasive technique, the application of LLR has increased rapidly. Initially, LMLR was mainly applied for HCCs located in anterolateral segments (18, 19). With the development of laparoscopic equipment and techniques, LMLR was gradually applied in difficult segments and LMH were also gradually applied for HCCs. However, the oncological adequacy of LLR for HCCs, especially LMLR-DS and LMH, has become an important topic of debate. Although several reports of the successful outcomes of LMLR-DS and LMH for HCCs have been published (20–23), most of them were performed with small number of cases, and the long-term outcomes were not available in most studies. Our present study included a large number of HCC patients who received LLR. The data of a relative long follow-up period were also included. Besides, we employed PSM to decrease the inter-group baseline differences. Moreover, subgroup analysis of either LMLR-DS or LMH for HCCs was performed, respectively. In these respects, our study was meaningful and convincing.

The Asia Pacific Consensus and Southampton Consensus Guidelines indicate that more evidence is needed to support the growth of LMLR-DS and LMH for HCCs (6, 7). One major problems of LMLR in segment 7 or 8 is the limited visualization (24), and several measures might be useful for overcoming this limitation in our experience: (1) the patient lied down with a cushion underneath the right back; (2) the right and cephalic sides of the operating table were raised; (3) the whole liver ligaments were separated and then the assistant pushed the right liver toward the left anterior inferior direction; (4) a water balloon was pulled under the right diaphragm to elevate the right posterosuperior segment. For LMLR in segment 1, the operative approach has always been an important issue. Three surgical approaches are described: left side, right side and trans-parenchymal approach (25, 26). Left side approach is commonly used for HCC in Spiegel’s lobe. The most common approach for HCC in caudate process is from the right side. Our previous study showed that the trans-parenchymal approach might be suitable for selected HCC originating in the paracaval portion (27). Another major problem of LMLR-DS is the intraoperative bleeding. As the difficult segments (1, 4a, 7, 8) were close to the main hepatic veins, skilled suture techniques are necessary for control of hepatic vein bleeding. 3D laparoscopic system, which offers the surgeon binocular vision and depth perception, might be benefit for suture compared with 2D system (28). Together, by using the above measures, LMLR-DS for HCCs could be successfully performed in most cases.

Most of the HCCs were combined with liver cirrhosis (29). LMH for HCCs is technically more demanding because of the increased risk of intraoperative bleeding and postoperative liver failure, especially in cirrhotic patients (22, 30). Some measures might be benefit for its performance in our experience. Firstly, Preoperative evaluation of liver function, Child-Pugh Grade A and ICG-R15 less than 10%, and remaining liver volume that accounts for >40% might be the prerequisites for HCC patients with liver cirrhosis to receive LMH (31, 32). Secondly, priority of Glissonian pedicle ligation could help to control the intraoperative bleeding (33), and Laennec’s approach is a recently reported easy and reliable measure to isolate the Glissonean pedicles (34). The Laennec’s approach might be more easily performed under the laparoscopic system because of the visual amplification compared with open surgery. Thirdly, the anterior approach of major hepatectomy, with benefit of reducing intraoperative bleeding compared with conventional approach, might be more easily performed under the laparoscopic system (35, 36). Fourthly, Pringle maneuver and low central venous pressure (LCVP, less than 5 cmH_2_O) was used to reduce intraoperative bleeding (37–39). Through the above measures, LMH for HCCs, especially with liver cirrhosis, could be successfully performed in most cases.

In our study, the short-term outcomes of LLR for HCCs were analyzed. In general, LLR had significantly less intraoperative blood loss and less blood transfusion compared with OLR. The control of hepatic vein bleeding is the major issue for LR. Although LCVP was performed in both LLR and OLR, the combination of LCVP and high pneumoperitoneum pressure (HPP, 13mmHg) might be more useful for control of hepatic vein bleeding in LLR. With the visual magnification of laparoscopic system, the parenchyma dissection in LLR might be more precise compared with it in OLR, which might be benefit for the decrease of intraoperative bleeding. Besides, the overall and severe postoperative complications after LLR were less than them after OLR. Because of the lower rate of complications, the postoperative hospital stays after LLR were shorter than it after OLR. Moreover, subgroup analyses confirmed that LMLR-DS had better short-term outcomes than OMLR-DS for HCCs. Patients after LHM also had lower operative blood loss and shorter postoperative hospital stays than OHM. Taken together, LLR could be a safe measure for HCC treatment.

The most important point of this study was to assess the long-term outcomes of LLR for HCCs. In general, LLR had comparable OS and DFS with OLR for HCCs. In subgroup analyses, LMLR-DS had similar OS and DFS with OMLR-DS for HCCs, and consistently, the OS and DFS had no differences between LMH and OMH groups. Taken together, considering that LLR had comparable long-term outcomes with OLR, LLR could be a reliable measure for HCC treatment. Thus, the indications of laparoscopic approach for HCCs in our center were mainly consistent with those of open approach. Besides, several points should be noticed for improving the long-term outcomes of either LLR or OLR for HCCs, which need to be validated further. Firstly, a wide-margin LR (≥1cm) might improve the long-term outcomes for patients with HCC (40, 41). Secondly, anatomic LR might be superior to non-anatomic LR regarding the long-term outcomes for HCCs (42, 43). Thirdly, the anterior approach for major hepatectomy with large HCC might have better long-term outcomes compared with the conventional approach (44).

Although our study included a large number of patients who received LLR for HCC, it still had limitations because that it was a retrospective study. Hence, a well-designed prospective study will be needed to affirm the validity of LLR for HCCs.

## Conclusions

Our results indicated that LLR for HCCs showed better short-term outcomes and comparable long-term outcomes with OLR, even for patients who received LMLR in difficult segments (1, 4a, 7 and 8) or LMH. Thus, LLR could be reliable and recommended for HCC treatment.

## Data availability statement

The original contributions presented in the study are included in the article/**Supplementary Material**. Further inquiries can be directed to the corresponding authors.

## Ethics statement

The studies involving human participants were reviewed and approved by Ethics Committee of Southwest Hospital of Army Medical University. The patients/participants provided their written informed consent to participate in this study. Written informed consent was obtained from the individual(s) for the publication of any potentially identifiable images or data included in this article.

## Author contributions

JL, SZ, FT, SL and JW participated in research design, data analysis, and writing of the manuscript. JC, YC, LC, XW and XL participated in data collection and analysis. All authors read and approved the final manuscript. All authors contributed to the article and approved the submitted version.
