# Supplementary material for: Long-term outcomes of laparoscopic liver resection versus open liver resection for hepatocellular carcinoma: A single-center 10-year experience
Source: Front Oncol. 2023 Jan 25;13:1112380. doi: 10.3389/fonc.2023.1112380 (PMC9905741; doi:10.3389/fonc.2023.1112380)
Supplement: Supplementary file 1 [file Table_1.doc]

**Supplementary Table S1. Number of patients with HCC in laparoscopic liver resection group (LLR) and open liver resection group (OLR).**

| **Year** | **Number of patients with HCC** | |
| --- | --- | --- |
|  | **LLR** | **OLR** |
| 2009 | 25 | 76 |
| 2010 | 23 | 45 |
| 2011 | 39 | 129 |
| 2012 | 60 | 140 |
| 2013 | 59 | 169 |
| 2014 | 86 | 161 |
| 2015 | 131 | 142 |
| 2016 | 128 | 117 |
| 2017 | 132 | 111 |
