# Supplementary material for: Long-term outcomes of laparoscopic liver resection versus open liver resection for hepatocellular carcinoma: A single-center 10-year experience
Source: Front Oncol. 2023 Jan 25;13:1112380. doi: 10.3389/fonc.2023.1112380 (PMC9905741; doi:10.3389/fonc.2023.1112380)
Supplement: Supplementary file 2 [file Table_2.doc]

**Supplementary Table S2** Baseline patient characteristics between laparoscopic minor liver resection in difficult segments (LMLR-DS) and open minor liver resection in difficult segments (OMLR-DS) groups. (*P < 0.05, statistical significance)

| **Characteristics** | **Before PSM** | | | **After PSM** | | |
| --- | --- | --- | --- | --- | --- | --- |
|  | **LMLR-DS（N=205）** | **OMLR-DS（N=391）** | **P** | **LMLR-DS（N=178）** | **OMLR-DS（N=178）** | **P** |
| **Age** | 51.00  (45.00-60.00) | 48.00  (42.00-57.00) | 0.001* | 51.00  (45.00-59.00) | 50.50  (45.00-60.00) | 0.857 |
| **Gender** |  |  | 0.341 |  |  | 1.000 |
| Male | 177 (86.3%) | 348 (89.0%) |  | 155 (87.1%) | 155 (87.1%) |  |
| Female | 28 (13.7%) | 43 (11.0%) |  | 23 (12.9%) | 23 (12.9%) |  |
| **Positive HBV-DNA** | 180 (87.8%) | 352 (90.0%) | 0.406 | 158 (88.8%) | 155 (87.1%) | 0.626 |
| **Positive HCV-RNA** | 2 (1.0%) | 5 (1.3%) | 0.744 | 2 (1.1%) | 2 (1.1%) | 1.000 |
| **Liver cirrhosis** | 139 (67.8%) | 268 (68.5%) | 0.854 | 123 (69.1%) | 122 (68.5%) | 0.909 |
| **Child-Pugh score** |  |  | 0.555 |  |  | 1.000 |
| A | 205 (100.0%) | 388 (99.2%) |  | 178 (100.0%) | 178 (100.0%) |  |
| B | 0 (0.0%) | 3 (0.8%) |  | 0 (0.0%) | 0 (0.0%) |  |
| **ASA score** |  |  | 0.608 |  |  | 0.831 |
| Ⅰ | 112 (54.6%) | 205 (52.4%) |  | 96 (53.9%) | 98 (55.1%) |  |
| Ⅱ | 93 (45.4%) | 186 (47.6%) |  | 82 (46.1%) | 80 (44.9%) |  |
| **TBIL(µmol/L)** | 14.60  (10.90-18.55) | 16.20  (12.80-20.20) | 0.001* | 15.35  (11.18-19.40) | 15.50  (12.40-19.05) | 0.402 |
| **ALT (IU/L)** | 33.00  (23.00-46.60) | 38.00  (27.00-55.30) | 0.002* | 33.05  (23.38-48.03) | 34.00  (25.00-52.00) | 0.252 |
| **ALB** | 42.70  (39.65-45.20) | 43.50  (40.50-46.10) | 0.035* | 42.80  (39.80-45.33) | 43.15  (40.18-45.70) | 0.520 |
| **PT (INR)** | 1.01  (0.97-1.04) | 1.03  (0.98-1.07) | 0.003* | 1.02  (0.97-1.05) | 1.01  (0.96-1.05) | 0.170 |
| **Platelet count**  **(*103 /μL)** | 128.00  (93.50-163.00) | 139.00  (101.00-183.00) | 0.006* | 129.50  (95.75-163.25) | 129.00  (92.75-162.75) | 0.773 |
| **AFP (≥400 ng/ mL )** | 45 (22.0%) | 149 (38.1%) | ＜0.001* | 41 (23.0%) | 46 (25.8%) | 0.537 |
| **ICG-R15(%)** | 4.60  (2.85-7.05) | 4.50  (2.70-7.20) | 0.726 | 4.60  (2.80-7.13) | 4.30  (2.58-7.25) | 0.431 |
| **Tumor number** |  |  | 0.761 |  |  | 0.814 |
| 1 | 193 (94.1%) | 366 (93.6%) |  | 169 (94.9%) | 168 (94.4%) |  |
| 2-3 | 12 (5.9%) | 24 (6.1%) |  | 9 (5.1%) | 10 (5.6%) |  |
| ≥4 | 0 (0.0%) | 1 (0.3%) |  |  |  |  |
| **Largest tumor diameter** |  |  | ＜0.001* |  |  | 1.000 |
| ≤5cm | 173 (84.4%) | 238 (60.9%) |  | 147 (82.6%) | 147 (82.6%) |  |
| ＞5cm | 32 (15.6%) | 153 (39.1%) |  | 31 (17.4%) | 31 (17.4%) |  |
| **Type of LR** |  |  | 0.444 |  |  | 0.586 |
| Anatomical LR | 81 (39.5%) | 142 (36.6%) |  | 71 (39.9%) | 66 (37.1%) |  |
| Non-anatomical LR | 124 (60.5%) | 249 (63.7%) |  | 107 (60.1%) | 112 (62.9%) |  |
| **Resection tumor margin** |  |  | 0.111 |  |  | 0.759 |
| ≥1cm | 199 (97.1%) | 368 (94.1%) |  | 172 (96.6%) | 173 (97.2%) |  |
| ＜1cm | 6 (2.9%) | 23 (5.9%) |  | 6 (3.4%) | 5 (2.8%) |  |
| **Margin status** |  |  | 1.000 |  |  | 1.000 |
| Negative | 205 (100.0%) | 390 (99.7%) |  | 178 (100.0%) | 178 (100.0%) |  |
| Positive | 0 (0.0%) | 1 (0.3%) |  | 0 (0.0%) | 0 (0.0%) |  |
| **Histological grade** |  |  | 0.059 |  |  | 0.731 |
| Low | 35 (17.1%) | 99 (25.3%) |  | 33 (18.5%) | 36 (20.2%) |  |
| Moderate | 155 (75.6%) | 271 (69.3%) |  | 134 (75.3%) | 128 (71.9%) |  |
| High | 15 (7.3%) | 21 (5.4%) |  | 11 (6.2%) | 14 (7.9%) |  |
| **Satellite nodule** |  |  | 0.170 |  |  | 1.000 |
| Positive | 0 (0.0%) | 5 (1.3%) |  | 0 (0.0%) | 0 (0.0%) |  |
| Negative | 205 (100.0%) | 386 (98.7%) |  | 178 (100.0%) | 178 (100.0%) |  |
| **Portal vein invasion** |  |  | 0.042* |  |  | 1.000 |
| Positive | 2 (1.0%) | 16 (4.1%) |  | 2 (1.1%) | 2 (1.1%) |  |
| Negative | 203 (99.0%) | 375 (95.9%) |  | 176 (98.9%) | 176 (98.9%) |  |
| **Bile duct invasion** |  |  | 1.000 |  |  | 1.000 |
| Positive | 0 (0.0%) | 0 (0.0%) |  | 0 (0.0%) | 0 (0.0%) |  |
| Negative | 205 (100.0%) | 391 (100.0%) |  | 178 (100.0%) | 178 (100.0%) |  |
| **TNM stage** |  |  | 0.055 |  |  | 0.759 |
| I-II | 199 (97.1%) | 365 (93.4%) |  | 173 (97.2%) | 172 (96.6%) |  |
| III-IV | 6 (2.9%) | 26 (6.6%) |  | 5 (2.8%) | 6 (3.4%) |  |

HBV, hepatitis B virus; HCV, hepatitis C virus; ASA American Society of Anesthesiologists;TBIL, total bilirubin; ALT, alanine transaminase; PT, prothrombin time; AFP,alpha-fetoprotein; ICG-R15, indocyanine green retention test at 15 minutes. *P < 0.05.
