# Supplementary material for: Long-term outcomes of laparoscopic liver resection versus open liver resection for hepatocellular carcinoma: A single-center 10-year experience
Source: Front Oncol. 2023 Jan 25;13:1112380. doi: 10.3389/fonc.2023.1112380 (PMC9905741; doi:10.3389/fonc.2023.1112380)
Supplement: Supplementary file 3 [file Table_3.doc]

**Supplementary Table S3** Baseline patient characteristics between laparoscopic major hepatectomy (LMH) and open major hepatectomy (OMH) groups.(*P < 0.05, statistical significance)

| **Characteristics** | **Before PSM** | | | **After PSM** | | |
| --- | --- | --- | --- | --- | --- | --- |
|  | **LMH**  **（N=124）** | **OMH**  **（N=380）** | **P** | **LMH**  **（N=115）** | **OMH**  **（N=115）** | **P** |
| **Age** | 49.91±1.050 | 48.59±0.55 | 0.245 | 49.69±1.10 | 52.17±1.05 | 0.105 |
| **Gender** |  |  | 0.380 |  |  | 0.370 |
| Male | 102 (82.3%) | 325 (85.5%) |  | 94 (81.7%) | 99 (86.1%) |  |
| Female | 22 (17.7%) | 55 (14.5%) |  | 21 (18.3%) | 16 (13.9%) |  |
| **Positive HBV-DNA** | 107 (86.3%) | 344 (90.5%) | 0.182 | 99 (86.1%) | 102 (88.7%) | 0.551 |
| **Positive HCV-RNA** | 0 (0%) | 4 (1.1%) | 0.576 | 0 (0.0%) | 0 (0.0%) | 1.000 |
| **Liver cirrhosis** | 77 (62.1%) | 254 (66.8%) | 0.334 | 73 (63.5%) | 77 (67.0%) | 0.580 |
| **Child-Pugh score** |  |  | 0.650 |  |  | 1.000 |
| A | 123 (99.2%) | 375 (98.7%) |  | 114 (99.1%) | 114 (99.1%) |  |
| B | 1 (0.8%) | 5 (1.3%) |  | 1 (0.9%) | 1 (0.9%) |  |
| **ASA score** |  |  | 0.133 |  |  | 0.791 |
| Ⅰ | 70 (56.5%) | 185 (48.7%) |  | 64 (55.7%) | 62 (53.9%) |  |
| Ⅱ | 54 (43.5%) | 195 (51.3%) |  | 51 (44.3%) | 53 (46.1%) |  |
| **TBIL(µmol/L)** | 15.4  (12.10-18.50) | 16.40  (12.55-20.20) | 0.025* | 15.7  (12.50-18.70) | 14.80  (11.70-14.80) | 0.832 |
| **ALT (IU/L)** | 35.00  (24.93-51.00) | 39.95  (27.00-58.00) | 0.041* | 35.40  (26.60-51.00) | 34.80  (23.00-49.00) | 0.402 |
| **ALB** | 41.79±0.36 | 42.28±0.22 | 0.245 | 41.90±0.38 | 41.85±0.39 | 0.916 |
| **PT (INR)** | 1.02  (0.96-1.07) | 1.03  (0.98-1.08) | 0.033* | 1.03  (0.97-1.07) | 1.01  (0.96-1.06) | 0.420 |
| **Platelet count**  **(*103 /μL)** | 159.50  (113.00-205.00) | 160.00  (118.25-218.00) | 0.470 | 159.00  (109.00-205.00) | 160.00  (124.00-222.00) | 0.329 |
| **AFP(≥400 ng/ mL )** | 42 (33.9%) | 166 (43.7%) | 0.054 | 41 (35.7%) | 39 (33.9%) | 0.782 |
| **ICG-R15(%)** | 3.80  (2.43-6.03) | 4.50  (2.50-6.78) | 0.064 | 3.70  (2.40-5.80) | 4.00  (2.70-6.30) | 0.329 |
| **Tumor number** |  |  | 0.631 |  |  | 0.881 |
| 1 | 105 (84.7%) | 334 (87.9%) |  | 98 (85.2%) | 96 (83.5%) |  |
| 2-3 | 17 (13.7%) | 42 (11.0%) |  | 15 (13.0%) | 16 (13.9%) |  |
| ≥4 | 2 (1.6%) | 4 (1.1%) |  | 2 (1.7%) | 3 (2.6%) |  |
| **Largest tumor diameter** |  |  | ＜0.001* |  |  | 0.894 |
| ≤5cm | 60 (48.4%) | 106 (27.9%) |  | 51 (44.3%) | 50 (43.5%) |  |
| ＞5cm | 64 (51.6%) | 274 (72.1%) |  | 64 (55.7%) | 65 (56.5%) |  |
| **Type of LR** |  |  | 0.147 |  |  | 0.895 |
| Anatomical LR | 70 (56.5%) | 186 (48.9%) |  | 64 (55.7%) | 63 (54.8%) |  |
| Non-anatomical LR | 54 (43.5%) | 194 (51.1%) |  | 51 (44.3%) | 52 (45.2%) |  |
| **Resection tumor margin** |  |  | 0.484 |  |  | 0.581 |
| ≥1cm | 118 (95.2%) | 355 (93.4%) |  | 109 (94.8%) | 107 (93.0%) |  |
| ＜1cm | 6 (4.8%) | 25 (6.6%) |  | 6 (5.2%) | 8 (7.0%) |  |
| **Margin status** |  |  |  |  |  | 1.000 |
| Negative | 124（100.0%） | 375（98.7%） |  | 115 (100.0%) | 115 (100.0%) |  |
| Positive | 0 (0.0%) | 5 (1.3%) |  | 0 (0.0%) | 0 (0.0%) |  |
| **Histological grade** |  |  | 0.008* |  |  | 0.228 |
| Low | 11 (8.9%) | 80 (21.1%) |  | 11 (9.6%) | 17 (14.8%) |  |
| Moderate | 106 (85.5%) | 285 (75.0%) |  | 99 (86.1%) | 89 (77.4%) |  |
| High | 7 (5.6%) | 15 (3.9%) |  | 5 (4.3%) | 9 (7.8%) |  |
| **Satellite nodule** |  |  | 0.088 |  |  | 1.000 |
| Positive | 5 (4.0%) | 33 (8.7%) |  | 5 (4.3%) | 5 (4.3%) |  |
| Negative | 119（96.0%） | 347（91.3%） |  | 110 (95.7%) | 110 (95.7%) |  |
| **Portal vein invasion** |  |  | ＜0.001* |  |  | 0.525 |
| Positive | 14 (11.3%) | 107 (28.2%) |  | 14 (12.2%) | 11 (9.6%) |  |
| Negative | 110 (88.7%) | 273 (71.8%) |  | 101 (87.8%) | 104 (90.4%) |  |
| **Bile duct invasion** |  |  | 0.572 |  |  | 1.000 |
| Positive | 1 (0.8%) | 2 (0.5%) |  | 1 (0.9%) | 0 (0.0%) |  |
| Negative | 123 (99.2%) | 378 (99.5%) |  | 114 (99.1%) | 115 (100.0%) |  |
| **TNM stage** |  |  | ＜0.001* |  |  | 0.478 |
| I-II | 103 (83.1%) | 252 (66.3%) |  | 94 (81.7%) | 98 (85.2%) |  |
| III-IV | 21 (16.9%) | 128 (33.7%) |  | 21 (18.3%) | 17 (14.8%) |  |

HBV, hepatitis B virus; HCV, hepatitis C virus; ASA American Society of Anesthesiologists;TBIL, total bilirubin; ALT, alanine transaminase; PT, prothrombin time; AFP,alpha-fetoprotein; ICG-R15, indocyanine green retention test at 15 minutes. *P < 0.05.
